# Conjuring up creativity: the effect of performing magic tricks on divergent thinking

**DOI:** 10.7717/peerj.11289

**Published:** 2021-04-15

**Authors:** Richard Wiseman, Amy Wiles, Caroline Watt

**Affiliations:** 1 School of Psychology and Sport Science, University of Hertfordshire, Hatfield, Hertfordshire, United Kingdom; 2 Unaffiliated, London, United Kingdom; 3 School of Philosophy, Psychology and Language Sciences, University of Edinburgh, Edinburgh, Midlothian, United Kingdom

**Keywords:** Magic, Psychology, Creativity, Divergent thinking, Conjuring, Self-esteem, Children

## Abstract

Research suggests that learning to perform magic tricks can promote both physical and psychological wellbeing. The current study extended this work by examining the impact of learning magic tricks on divergent thinking. A group of 10- to 11-year-old children completed Guilford’s Alternate Uses Test both before and after participating in either a magic-based, or art-based, activity. As predicted, compared to the art-based activity, the magic-based activity resulted in a significantly greater increase in both AUT Fluency and AUT Originality scores. Rosenberg’s Self-Esteem Scale and Dweck’s Implicit Theories of Intelligence Scale for Children was also completed after each activity, and participants’ self-esteem scores were higher after the art-based activity than the magic-based activity. In an exploratory aspect of the study, the AUT was re-administered to both groups three weeks later, and yielded no significant differences. The practical and theoretical implications of these findings are discussed, along with recommendations for future research.

## Introduction

Research suggests that learning to perform magic tricks can result in a range of psychological and physical benefits, including improved fine and gross motor movements, enhanced social skills and raised self-esteem (for reviews, see Lam, Lam & Chawla, 2017; Wiseman & Watt, 2018; Bagienski & Kuhn, 2019). Similar magic-based interventions can also have a positive impact within a pedagogical context, and have been used to promote science education, increase curiosity and heighten critical thinking skills (for a review, see Wiseman & Watt, 2020). The current study builds on this work by examining whether learning to perform magic tricks also enhances children’s divergent thinking.

Showing someone how to perform a magic trick involves two main stages. In the first stage, the person sees a sequence of events in which their expectations about causation are violated. For instance, a performer might show a box to be empty, wave a magic wand and produce a bowling ball from the box. Magicians refer to this as the ‘effect.’ In the second stage, the performer revealed the secret to the illusion. For instance, the performer might explain how the box contained several cleverly placed mirrors that concealed the bowling ball. Magicians refer to this as the ‘method.’ Divergent thinking is usually seen as the ability to create many different solutions to a problem or issue, and several strands of research suggest that such thinking may be enhanced by seeing magic tricks and/or learning the secrets to these tricks. Each of these strands of work will be discussed in turn.

In terms of watching a magic effect, some researchers have explored the impact of experiencing events that violate expectations or appear impossible. According to this approach, people perceive and understand the world using mental models (referred to as ‘schemata’), and the reliance on such schemata during problem solving results in the production of more predictable and traditional solutions. Similarly, any activity that disrupts or disconfirms such schemata will help individuals to generate more novel and original ideas. The results from several strands of research confirm this notion.

First, some researchers have examined the effect that disrupting people’s stereotypical thinking about social categorisations has on their divergent thinking. For instance, Vasiljevic & Crisp (2013) asked participants to generate either stereotypical social categories (e.g., ‘poor student’) or non-stereotypical categories (e.g., ‘rich student’), and then complete a test of divergent thinking. Those who had generated the non-stereotypical social categories obtained higher scores. Similarly, Gocłowska & Crisp (2013) had participants generate adjectives to describe either a stereotypical target (e.g., a male mechanic) or non-stereotypical target (e.g., a female mechanic), and then complete the Guilford Alternate Uses Test (AUT: Guilford, 1967). The AUT is one of the most widely used measures of divergent thinking, and involves participants attempting to generate alternative uses for everyday objects. Those working with the non-stereotypical targets generated a greater number of more original ideas.

Second, developmental psychologists have shown a positive relationship between children’s divergent thinking and their preference for fantasy-orientated play (i.e., play involving magical powers, impossible events, or imaginary companions). For instance, Russ & Grossman-McKee (1990) reported that the degree to which children appeared to enjoy fantasy play was positively correlated with their AUT scores. In a longitudinal study, Russ, Robins & Christiano (1999) showed that children’s preference for fantasy play was correlated with divergent thinking over a 4-year period. More recently, Bunce & Woolley (2021) found a positive correlation between children’s preference for fantasy play, and measures of both verbal and physical creativity.

Third, other work has examined whether people become more divergent thinkers when they experience magical, fantastical or other ‘impossible’ events. Subbotsky, Hysted & Jones (2010) had children complete a divergent thinking test, watch a short film that either featured ‘magical’ events (e.g., talking animals or wizards performing the impossible) or involved the same characters performing non-magical actions, and then completed the test a second time. The children who viewed the ‘magical’ film clip subsequently obtained significantly higher divergent thinking scores than those watching the ‘non-magical’ film clip. Similarly, Ritter et al. (2012) placed students into a virtual environment and had them experience either ‘magical’ events (e.g., a suitcase that inexplicably changed size and a bottle that levitated) or non-magical events, and then complete the AUT. Participants who had experienced the ‘magical’ events exhibited significantly higher divergent thinking scores.

The above work suggests that witnessing a magic trick may help to boost divergent thinking. In addition, discovering the method of a trick may also foster divergent thinking. Past work suggests that being exposed to examples of divergent thinking enhances creativity (for reviews, see Scott, Leritz & Mumford, 2004a, 2004b), and several studies have demonstrated that discovering the secret to some magic tricks involves divergent thinking. For instance, Danek et al. (2014) showed that solving magic tricks resulted in the same type of ‘a-ha’ experience associated with suddenly figuring out the solutions to insight problems. Similarly, Hedne, Norman & Metcalfe (2016) explored metacognitive awareness in insight problem solving by asking participants to rate how close they felt to a solution whilst attempting to solve a series of magic tricks. Thomas & Didierjean (2016) also showed how presenting participants with false solutions to magic tricks prevented the type of creative thinking necessary to solve the trick. This work is supported by an MRI study conducted by Parris et al. (2009), in which participants’ brain activity was monitored while they watched a magic trick. The results suggested that watching the trick activated the anterior cingulate cortex, which is associated with processing anomalous information (Fugelsang & Dunbar, 2005) and cognitive flexibility (Leber, Turk-Browne & Chun, 2008).

In addition to this empirical work, educational practitioners and researchers have presented anecdotal evidence to suggest that learning magic tricks boosts divergent thinking and several organisations have employed such tricks in creativity workshops (e.g., Vidler & Levine, 1981; McCormack, 1985, 1990; Tognazzini, 1993; Hepworth, 2007).

Given the large body of work suggesting a relationship between learning to perform magic tricks and divergent thinking, it’s perhaps surprising that very few experimental studies have explored the topic. Haritaipan, Saijo & Mougenot (2018a) showed novice design students descriptions of different kinds of magical illusions and then asked them to create a novel form of mug. Compared to a group that hadn’t seen the descriptions, the students produced more creative designs. A follow up study showed that this effect was enhanced when the students saw both the illusions and the types of methods often used by magicians to create these tricks (Haritaipan, Saijo & Mougenot, 2018b). Most recently, Li (2020) conducted a study with students enrolled on a course about the design and development of software applications. Participants completed the AUT and then took part in a five-stage, magic-based, intervention that was designed to boost divergent thinking. This involved them producing solutions to a simple problem (e.g., how do you transfer a ring from one finger to another), seeing a trick that seemed to present a magical solution to the problem, considering possible solutions to the trick and discovering how it was achieved, performing the trick for one another, and coming up with creative solutions to other problems. Participants were interviewed after the intervention and completed the AUT a second time. A thematic analysis revealed that participants believed that the process had helped them to develop a childlike perspective, promoted curiosity, and helped to boost creative problem solving. Although the study didn’t involve a control group, participants’ post-intervention AUT scores were significantly higher than their pre-intervention scores.

Although these results are promising, it’s difficult to draw strong conclusions as the studies were conducted with specific cohorts (design students) and/or often did not involve appropriate control conditions. The current study began to address these issues by exploring whether school children’s divergent thinking skills were increased by them learning magic tricks, and comparing the magic-based intervention with a matched art-based activity. In the study, children first completed the AUT, participated in either a magic-based or art-based intervention, and then completed the AUT a second time. Participants’ AUT responses were blind coded for both Fluency (number of ideas) and Originality (novelty of ideas). These two scales were chosen because they reflected key processes involved in the creation of magic tricks. It was predicted that both sets of post-intervention scores would be significantly higher after the magic-based activity than the art-based activity.

A secondary aspect of the study examined whether learning magic tricks may boost self-esteem and a growth mindset. Due to the time restraints, these measures were only administered post-intervention and thus provided limited insights.

Some researchers have speculated that the sense of mastery involved in learning and presenting a magic trick might boost self-esteem (e.g., Ezell & Klein-Ezell, 2003). Previous research into this notion has obtained mixed results. For instance, Ezell & Klein-Ezell (2003) taught magic tricks to physically and psychologically challenged children, and obtained increased scoring on The Student Self-Concept Scale. In a similar study involving severely emotionally disturbed children, Levin (2007) informally reported increases on Rosenberg’s (1965) Self-Esteem Scale (SES), but did not carry out any statistical analysis of their data. Other researchers have failed to find such effects. Kwong (2007) reported that a magic-based intervention did not significantly boost hospital inpatients’ SES scores, and Sui & Sui (2007) did not obtain an overall significant increase in patients’ scores on the Chinese General Self-Efficacy Scale. The current study added to this work by examining whether the magic-based and art-based interventions impacted on participants’ SES scores.

Dweck’s (2000, 2006) ‘mindset’ theory posits that people vary in the degree to which they believe that their abilities and skills are either malleable (‘Growth Mindset’) or unchangeable (‘Fixed Mindset’). A large body of work suggests that a Growth Mindset is associated with higher levels of resilience and achievement, and that certain activities help people moved from a Fixed Mindset to a Growth Mindset (for reviews, see Burnette et al., 2013: Dweck & Yeager, 2019). Given that learning how to perform a magic trick involves the acquisition of both new information and skills, it seems plausible that it might help to develop a growth mindset. To our knowledge, no previous work has examined how learning magic tricks might impact on mindset. The current study examined this issue by having participants complete Dweck’s (2000) ‘Implicit Theories of Intelligence Scale for Children’ (ITISC).

Finally, a few weeks after the formal study, an opportunity arose to have participants complete the AUT, SES and ITISC again. Data from the two conditions were compared, but no predictions were made about these exploratory analyses and the results are reported for completeness.

## Method

### Participants

The study involved children in two classes of a North London Primary School. All children enrolled in Year 6 were invited to participate, with 63 returning written parental consent (Age: 10–11 years old: 26 male, 35 female, 2 no response). Six participants did not take part in the exploratory session (*N* = 57: 25 male, 31 female, 1 no response).

### Design

The study employed a 2 (Intervention: ‘Magic’ and ‘Art’) × 2 (Time: ‘Pre-intervention’ and ‘Post-intervention’) mixed design, with two dependent variables (AUT Fluency and AUT Originality scores). Participants also completed the SES and ITISC during the post-intervention period, and during the third, exploratory, session they completed the AUT, the ITISC and SES. Due to the lack of previous research on this topic, it wasn’t possible to estimate an expected effect size in advance of the experiment. However the sample size had a high chance of detecting a medium effect (d = 0.5, *p* < 0.05, 2-tailed, power = 0.8). No other measures were administered or data collected.

### Materials

#### Magic intervention

Due to time constraints, the children were only taught one magic trick. This trick is easily available from magic stores and has various names, including ‘Die Vision’ and ‘Colour Vision.’ This trick was chosen because it was economical to purchase, fooling, relatively easy to master and the solution could be figured out via divergent thinking. A researcher first showed participants a simple magic trick in which a child was given a cube with different coloured faces, and asked to place the cube into a small box. Despite not being able to see into the box, the researcher was able to tell which colour was facing upwards. Participants were given several minutes to discuss the trick in groups and offer suggestions about how it was done. Participants were then shown the secret of the trick, provided with the apparatus and asked to practice the trick in pairs. Finally, they were given the opportunity to perform the trick in front of the class.

#### Art intervention

A researcher showed participants how to make a one-point perspective drawing. This involved them drawing a horizon line and vanishing point, and then using this set-up to draw a road that appeared to go into the distance. Participants were shown how the same technique could be used to draw a railway track, a river and a tunnel. Finally, participants were then provided with a paper template, shown how to use it and asked to create their own perspective pictures. This activity was created to share many of the features associated with the magic-based intervention, including being participatory, fun, visual, interesting, counter-intuitive and encouraging a sense of self-mastery. However, it did not involve any trickery, or generate a seemingly impossible event or experience.

#### *Alternate uses test* (AUT: Guilford, 1967)

During the formal part of the study, participants were presented with a drawing of either a skipping rope or a mug, and given 3 min to note down as many uses for the object as possible. The two drawings were counterbalanced across the Pre-intervention and Post-intervention periods for each participant. During the third test session, all participants were given 3 min to produce as many uses as possible for a ruler.

Two independent blind raters counted the number of ideas listed on each AUT response sheet (Fluency) and rated the overall novelty of the ideas on a 7-point scale between ‘1: Not very creative’ to ‘7: Very creative’ (Originality). All inter-rater reliabilities were high and statistically significant (Pre-intervention (*N* = 63): Fluency r = 0.99, *p* < 0.001; Originality r = 0.91, *p* < 0.001 Post-intervention (*N* = 63): Fluency r = 1, *p* < 0.001; Originality r = 0.88, *p* < 0.001 Third-session (*N* = 57): Fluency r = 1, *p* < 0.001, Originality r = 0.91, *p* < 0.001). Fluency and Originality measures were obtained for each participant by averaging the two raters’ scores.

#### *Self-esteem scale* (SES: Rosenberg, 1965)

Participants were presented with 10 statements about self-esteem (e.g., ‘I feel I have a number of good qualities’) and indicated their level of agreement to each statement on a scale between 1 (strongly disagree) and 4 (strongly agree). Higher scores reflected higher levels of self-esteem.

#### *Implicit theories of intelligence scale for children* (ITISC: Dweck, 2000)

Participants were presented with three statements about whether they believe their intelligence is fixed (e.g., ‘You have a certain amount of intelligence and you can’t do much to change it’) and indicated their level of agreement to each statement on a scale between 1 (strongly agree) and 6 (strongly disagree). Higher scores reflected higher levels of a growth mindset.

### Procedure

The study received ethics approval (number LMS/PGT/PGT/UH/02830) from the University of Hertfordshire Research Ethics Committee. The study was conducted during school hours in lieu of a regular lesson. Participants were randomly assigned to the two conditions (with the occasional exception to avoid timetable clashes). Participants completed the AUT (‘Pre-Intervention’), took part in either the magic or art intervention, and completed the AUT a second time (‘Post-Intervention’). During the post-intervention period, participants also completed the SES. An unexpected opportunity arose to carry out additional testing 17 days after the study, and during this third session participants completed both the AUT and the SES.

## Results

AUT Fluency (Table 1): A 2 × 2 mixed ANOVA showed a non-significant effect upon AUT Fluency scores of Intervention (*F* [1, 61] = 0.05, *p* = 0.82, eta squared = 0), a significant effect of Time (*F* [1, 61] = 5.01, *p* = 0.03, eta squared = 0.07) and a significant interaction (*F* [1, 61] = 7.23, *p* = 0.01, eta squared = 0.10).

**Table 1:** AUT fluency scores.

	Pre-intervention	Post-intervention	Total
**Magic intervention** (*N* = 31)	5.37 (2.14) [4.58–6.16]	7.10 (3.28) [5.89–8.30]	6.23 (2.34) [5.50–7.00]
**Art intervention** (*N* = 32)	6.45 (3.36) [5.24–7.66]	6.31 (2.76) [5.31–7.31]	6.38 (2.77) [5.62–7.14]
**Total**	5.92 (2.86) [5.20–6.64]	6.70 (3.03) [5.93–7.46]	

**Note:**

Means, standard deviations (parentheses), and 95% CI [square parentheses], of AUT Fluency scores by Intervention and Time.

Post hoc paired *t*-tests revealed that the pre to post scores were not significantly different for the Art Intervention (*t*_31_ = 0.30, *p*(2−t) = 0.77, *d* = 0.05), and that the post scores were significantly higher than the pre scores for the Magic Intervention (*t*_30_ = 3.38, *p*(2−t) = 0.002, *d* = 0.62).

AUT Originality (Table 2): A 2 × 2 mixed ANOVA showed a non-significant effect upon AUT Originality scores of Intervention (*F* [1, 61] = 1.13, *p* = 0.29, eta squared = 0.02), a non-significant effect of Time (*F* [1, 61] = 3.65, *p* = 0.06, eta squared = 0.06), and a significant interaction (*F* [1, 61] = 8.86, *p* = 0.004, eta squared = 0.12).

**Table 2:** AUT originality scores.

	Pre-intervention	Post-intervention	Total
**Magic intervention** (*N* = 31)	3.5 (1.24) [3.04–3.95]	4.26 (1.53) [3.70–4.82]	3.88 (1.26) [3.56–4.24]
**Art intervention** (*N* = 32)	4.31 (1.65) [3.73–5.00]	4.16 (1.42) [3.64–4.67]	4.23 (1.40) [3.80–4.60]
**Total**	3.91 (1.50) [3.54–4.29]	4.21 (1.50) [3.84–4.57]	

**Note:**

Means, standard deviations (parentheses), and 95% CI [square parentheses], of AUT Originality scores by Intervention and Time.

Post hoc paired *t*-tests revealed that the pre to post scores were not significantly different for the Art Intervention (*t*_31_ = 0.72, *p*(2−t) = 0.48, *d* = 0.1), and that the post scores were significantly higher than the pre scores for the Magic Intervention (*t*_30_ = 3.5, *p*(2−t) = 0.001, *d* = 0.55**)**.

### Secondary analyses

SES scores were significantly higher after the art intervention than the magic intervention (Magic *N* = 31, M = 29.32, SD = 4.27, CI [27.76–30.89]; Art *N* = 32, M = 32.41, SD = 4.51, CI [30.78–34.03]; *t*_61_ = 2.79, *p*(2−t) = 0.007, *d* = 0.70).

The ITISC scores were not significantly different between the two conditions (Magic *N* = 31, M = 4.35, SD = 0.75, CI [0.27–4.08]; Art *N* = 32, M = 4.50, SD = 1.08, CI [0.39–4.89]; *t*_61_ = 0.62, *p*(2−t) = 0.54, *d* = 0.19).

### Exploratory analyses

During the third testing period, there were no significant differences between the conditions in either AUT Fluency (Magic *N* = 26, M = 6.27, SD = 2.01, CI [5.46–7.08]; Art *N* = 31, M = 6.52, SD = 2.80, CI [5.49–7.54]; *t*_55_ = 0.37, *p*(2−t) = 0.71, *d* = 0.10) or AUT Originality (Magic *N* = 26, M = 4.19, SD = 1.62, CI [3.54–4.85]; Art *N* = 31, M = 4.48, SD = 1.77, CI [3.83–5.13]; *t*_55_ = 0.64, *p*(2−t) = 0.52, *d* = 0.17).

The SES scores remained significantly higher among those that had experienced the art intervention (Magic *N* = 26, M = 29.80, SD = 4.23, CI [28.08–31.53]; Art *N* = 31, M = 32.24, SD = 4.60, CI [30.55–33.93]; *t*_55_ = 2.06, *p*(2−t) = 0.04, *d* = 0.55).

The ITISC scores remained non-significant between the two conditions (Magic *N* = 26, M = 4.45, SD = 0.85, CI [0.34–4.10]; Art *N* = 31, M = 4.43, SD = 1.19, CI [0.49–3.99]; *t*_55_ = −0.06, *p*(2−t) = 0.95, *d* = 0.02).

## Discussion

This study examined whether learning to perform a magic trick helped to boost children’s divergent thinking. As predicted, compared to children participating in an art-based intervention, those learning a magic trick subsequently exhibited significantly higher AUT Fluency and Originality scores.

On a theoretical level, there are two main types of explanations that may account for the findings. A large body of work has shown that merely experiencing an event that disrupts existing schemata (including generating non-stereotypical social categories, engaging in fantasy play, or experiencing seemingly impossible events in a virtual environment) promotes divergent thinking (see, e.g., Ritter et al., 2012; Vasiljevic & Crisp, 2013; Bunce & Woolley, 2021). This work would suggest that witnessing the magic trick might account for the increase in AUT scores. In this study, participants saw a magic trick in which one person appeared to read another person’s mind. This demonstration may have violated participants’ existing schemata and beliefs about the privacy of thoughts, and resulted in them subsequently displaying greater levels of divergent thinking. However, other research has noted that many magic tricks are much like insight problems (see, e.g., Lamont & Wiseman, 1999; Kuhn, Amlani & Rensink, 2008; Rensink & Kuhn, 2015) and thus discovering the method of the trick may play a key role in promoting divergent thinking. In the current study, the method used to determine the colour selected during the trick is both clever and counter-intuitive. According to this perspective, discovering this clever solution may have enhanced their divergent thinking skills. Future work could examine these two competing explanations by, for instance, exploring whether divergent thinking is enhanced by observing magic tricks or must also involve discovering the secrets behind the illusions. Additional work could also examine whether a magic-based intervention also affects other aspects of participants’ responses on the AUT (e.g., flexibility and elaboration), and their scores on similar tests (e.g., The Torrance Test of Creative Thinking). Finally, in this study participants were taught a single trick that allowed them to apparently read someone’s mind. Future research could examine the impact of a greater diversity of magic tricks, including those that involve a far greater range of methods and effects.

On a more pragmatic level, learning magic tricks may provide an effective way of enhancing divergent thinking in the classroom and beyond. Educational organizations, researchers and writers have frequently emphasized the need to nurture divergent thinking from a young age (e.g., Torrance, 1977; Kimbell, 2000; Craft, 2005; Edwards, McGoldrick & Oliver, 2006; Kim, 2011; Robinson, 2015). Recent research in the area has focused on developing interventions that are relatively brief, practical to deliver and empirically based, including work examining the impact of adopting multiple perspectives (Gaither, Fan & Kinzler, 2019), playing certain types of video games (Blanco-Herrera, Gentile & Rokkum, 2019) and encouraging free expression (Moreau & Engeset, 2016). The type of magic-based intervention used in this study was relatively brief, easy to deliver, enjoyable, fitted into children’s current appetite for fantasy books and films, and was easily incorporated into a lesson format. Perhaps most promising of all, the study obtained these effects after participants had only learned and performed a relatively simple magic trick. Future work in this area could examine whether this impact could be magnified by children learning a greater number of more elaborate tricks, and whether this type of intervention is especially beneficial to certain types of participants (e.g., those with a prior interest in magic/performance or those that especially enjoy fantasy oriented play). This work could also examine whether any effect is limited to certain age groups and types of magic tricks. For example, Gopnik & Astington (1988) suggest that many children under the age of four struggle to appreciate many magic tricks because they have yet to fully establish a strong sense of natural causality.

These findings add to the growing literature exploring the positive impact that learning magic tricks can have on psychological wellbeing and thinking skills (see Bagienski & Kuhn, 2019; Wiseman & Watt, 2018, 2020). Wiseman & Watt (2018, 2020) noted that it is often problematic to draw strong conclusions from this research, as the studies have often tended to involve clinical populations and failed to employ control conditions. The current study involved a cohort of children and took place in an educational setting. In addition, it compared the effects of learning a magic trick with discovering how to make a one-point perspective drawing. This control condition was carefully created and possessed many of the features associated with the magic intervention, including being visual, interactive, enjoyable, interesting and encouraging a sense of self-mastery. It is hoped that this type of control condition will prove helpful to other researchers in this area, and may help to build a more systematic body of work from which it is possible to draw stronger conclusions.

Results from the secondary aspect of the study suggested that self-esteem was significantly higher among children who had participated in the art intervention compared to those who had learned how to perform a magic trick. However, this finding should be treated with caution as the data was only collected during the post-intervention period, and thus doesn’t provide a properly controlled measure of the potential impact of the interventions. Future research could follow up on this finding by measuring SES during both the pre-intervention and post-intervention periods. If this future work suggests that the effect is genuine, then it will clearly be important to understand the mechanisms at play. For instance, it could be that the art intervention provided a greater sense of mastery, or that presenting the magic trick to the class may have proved challenging for some children. This work could also investigate whether participants’ self-esteem was affected by whether they were able to solve the magic trick, with an inability to figure out the secret causing a lowering of self-esteem. Understanding such effects will clearly be important for the future development of magic-based interventions.

Results from the secondary aspect of the study also suggested that participant’s ITISC scores were not significantly different between the two conditions. However, again, there is good reason to treat this finding with caution. First, as noted above, the ITISC was only administered after the magic-based and art-based activities, and so may not have detected the impact of the interventions. Second, the ITISC measures whether participants believe that their intelligence is fixed or malleable, whereas the study involved learning new skills and abilities. Future research could address these issues by comparing mindset scores pre and post intervention, and employing a measure that reflects participants’ beliefs about their ability to change in general.

Finally, exploratory data collected three weeks after the formal study suggests that the boost to divergent thinking created by the magic-based intervention had dissipated. Again, this finding should be treated with caution because some participants were not involved in the additional testing session and the object used in the AUT wasn’t counter-balanced across participants. However, future work could examine whether this apparent decline is genuine and, if it is, whether magic-based interventions delivered over an extended period of time and involving several tricks, have a larger and longer-lasting effect.

## Conclusions

The findings from this study suggest that children participating in a magic-based intervention showed greater gains in divergent thinking compared to those participating in a matched art-based intervention. It adds to existing literature on the relationship between experiencing seemingly impossible events and divergent thinking, and the impact of learning magic tricks on wellbeing. It represents one of the few studies in the area to employ a control group and a cohort not involving design students. It also contributes to existing work exploring ways of boosting creativity in the classroom, and offers a relatively brief and highly practical way of increasing children’s divergent thinking. It is hoped that future research builds on these findings by examining the impact of different types of magic tricks, identifying the types of cohorts that find magic-based interventions especially beneficial, and exploring ways of increasing the longevity of the effect. If such work is successful, both educational practitioners and parents will have a novel and fascinating way to bring out the magic in children’s minds.

## Supplemental Information

10.7717/peerj.11289/supp-1 Supplemental Information 1 Dataset. The conditions and scores of each participant.

## Additional Information and Declarations

### Data Availability

The following information was supplied regarding data availability: The raw data are available in the Supplemental File.
